# Palpitations, headache and night sweats caused by a retroperitoneal mass: case report and short review

**DOI:** 10.1259/bjrcr.20170035

**Published:** 2017-08-02

**Authors:** Laura Wuyts, Marc Pouillon, Marc Huyghe, Natacha Ruyssers, Gerda Goovaerts, Mattias Spaepen

**Affiliations:** ^1^Department of Radiology, GZA Hospitals, Antwerp, Belgium; ^2^Department of General and Digestive Surgery, GZA Hospitals, Antwerp, Belgium; ^3^Department of Anatomical and Cellular Pathology, GZA Hospitals, Antwerp, Belgium

## Abstract

A 31-year-old West-African female attended our emergency department presenting with palpitations, headache, fatigue and night sweats during the last 2 weeks. Clinical examination revealed tachycardia and a painful, palpable infraumbilical mass. Ultrasound examination of the abdomen showed a smoothly rounded soft-tissue mass with a diameter of 5 cm. On contrast-enhanced CT, a prevertebral mass with intense contrast enhancement was seen, located caudal to the aortic bifurcation. On PET-CT, there were no distant ^18^F-FDG-avid locoregional nodes or masses. A tumourectomy was successfully performed, during which manipulation of the retroperitoneal tumour triggered a sharp rise in blood pressure. Histological analysis confirmed the diagnosis of a paraganglioma. The clinical complaints of headache, paroxysmal palpitations and night sweats disappeared postoperatively. This case is a classic presentation of a paraganglioma occurring in the organs of Zuckerkandl, a collection of paraganglia. The diagnosis should be suspected in the presence of a heterogeneous, hypervascular mass in the retroperitoneum and typical clinical symptoms of hypertension, headache and palpitations. Treatment involves surgical resection, after accurate preoperative management. Genetic counselling is required, allowing a personal and genotype-based follow-up.

## Clinical presentation

A 31-year-old female from West Africa was referred to the hospital by her general practitioner following a blood test showing inflammation and anaemia (CRP 352 mg l^–1^ and Hb 7.2 g dl^–1^). In the last 2 weeks, she had a sense of general malaise, fatigue and night sweats. Moreover, she also complained of palpitations and persistent headaches. In the emergency department, clinical examination revealed tachycardia and a painful, palpable infraumbilical mass.

## Imaging findings

**Figure 1.**
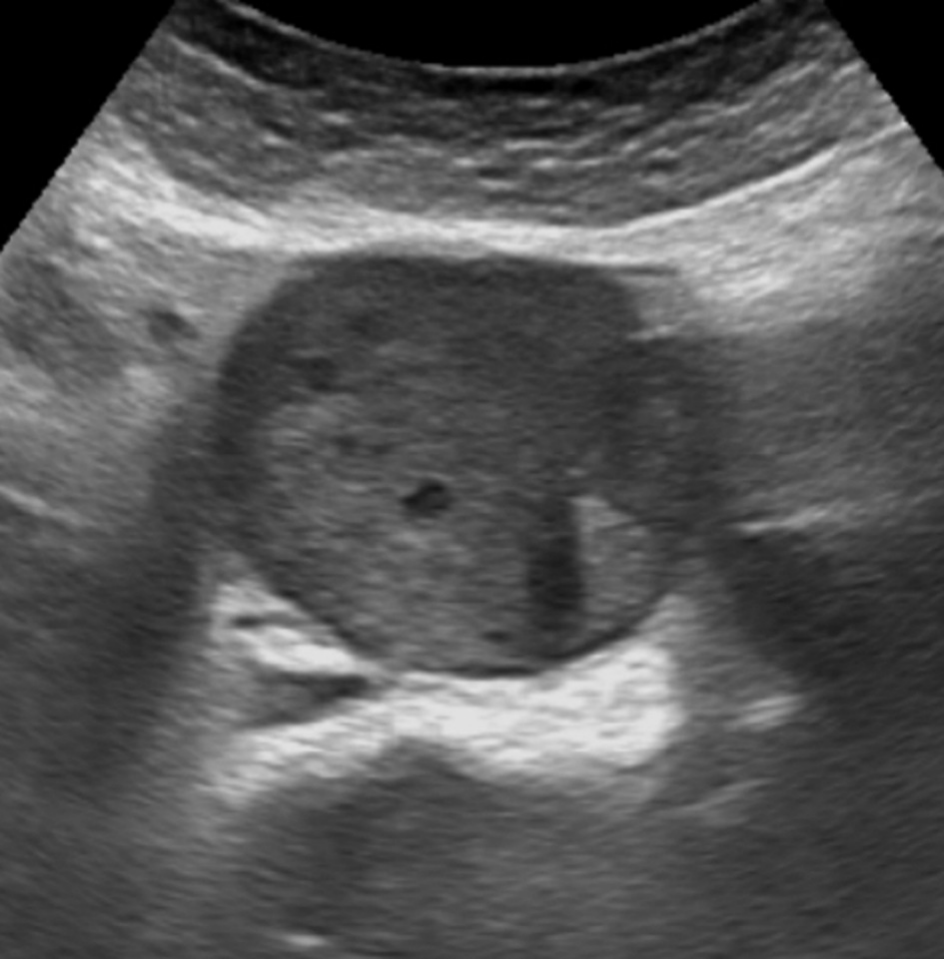
Abdominal ultrasound demonstrates a round soft-tissue mass with heterogeneous echogenicity and small areas of cystic degeneration.

**Figure 2.**
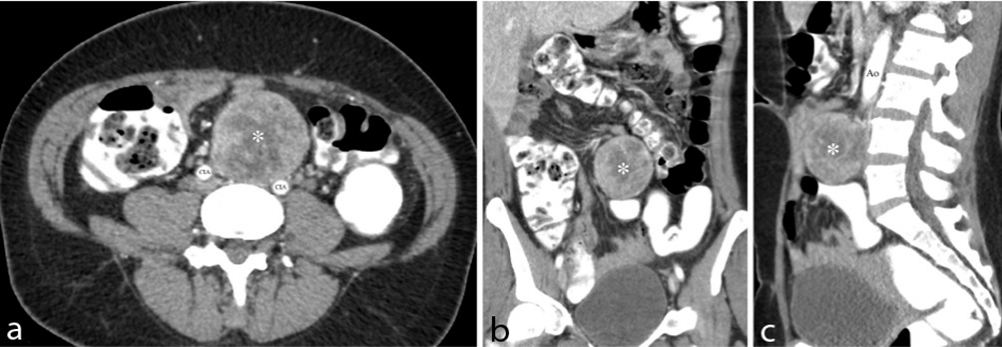
Contrast-enhanced CT with axial (a), coronal (b) and sagittal (c) reconstructions. Heterogeneous enhancing, well-defined soft-tissue mass (asterisk) located retroperitoneal caudal to the aortic bifurcation. There are no signs of an invasive growth pattern. Ao, aorta; CIA, common iliac artery.

Ultrasound examination of the abdomen demonstrated a prevertebral smoothly rounded soft-tissue mass with smooth edges and internal vascularization on colour Doppler. The mass was predominantly intermediate, reflective and had a diameter of 5 cm (Figure 1). A contrast-enhanced CT examination of the abdomen was performed in arterial and portal venous phase. A heterogeneously enhancing, nodular mass was seen caudal to the aortic bifurcation. There were no signs of an invasive growth pattern or distant metastases (Figure 2).

**Figure 3.**
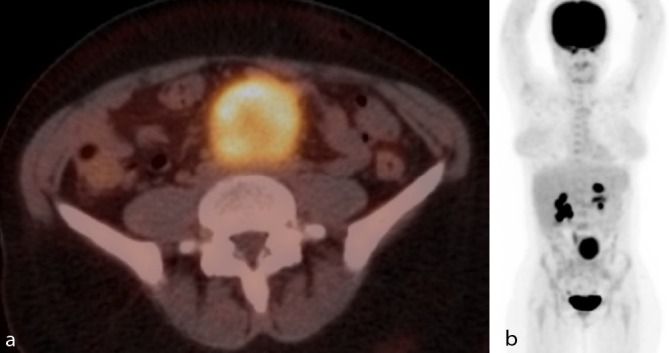
Axial ^18^F-FDG-PET CT image (a) and coronal maximal-intensity-projection ^18^F-FDG-PET image (b). There is a large, solitary and well-defined mass with marked avidity of ^18^F-fluorodeoxyglucose. There are no distant ^18^F-FDG-avid locoregional lymph nodes or masses.

To further differentiate and rule out metastatic disease, a positron emission tomography (PET)-CT was performed (Figure 3). One hour after intravenous injection of ^18^F-fluorodeoxyglucose (^18^F-FDG), inhomogeneous intense ^18^F-FDG uptake was seen in the lesion, with a high maximum standardized uptake value (SUV) of 12.2. There were no distant ^18^F-FDG-avid locoregional nodes or masses.

## Differential diagnosis

Based on the retroperitoneal location, the differential diagnosis is limited to neuroendocrine tumours (paraganglioma, schwannoma, neurofibroma), soft-tissue tumours and lymphadenopathy.

Because of the paravertebral location, neurogenic tumours must be considered. Among neurogenic tumours, schwannomas are the most common tumour of peripheral nerves. Although a schwannoma is also a well-defined mass, diagnosis is less likely because there is no continuity with the adjacent neural foramen. In case of a neurofibroma, we would expect a hypodense mass with only minimal or no contrast enhancement. This does not fit the presented case.

A liposarcoma, one of the most common primary retroperitoneal neoplasms, typically presents itself in a different way. A varying amount of fat and soft tissue, and multiple septae are expected. Leiomyosarcoma is another frequently encountered primary retroperitoneal sarcoma and large tumours have extensive areas of necrosis and occasional haemorrhage. Malignant fibrous histiocytomas are also large, moderately hypervascular, and heterogeneously enhancing soft-tissue masses with areas of necrosis and haemorrhage. However, we exclude both leiomyosarcoma and malignant fibrous histiocytoma due to the lack of invasion of adjacent organs. Lymphomas are presented as a lymph node mass. Due to their hypovascular nature, minimal contrast enhancement is seen. Often there is encasement of the large retroperitoneal vessels. Moreover, blood analysis can present associated abnormalities.

Given the age of the patient, the clinical presentation, and the morphology and location of the mass, the preferred diagnosis was a retroperitoneal paraganglioma.

## Treatment and follow-up

A laparotomy was performed to remove the lesion. The tumour was situated closely to the left ureter and left iliac artery, but there was no invasion. After ligation of the immediate vasculature, the well-defined tumour could be removed completely. During the operation, manipulation of the tumour triggered a sharp rise in blood pressure. After removal of the tumour, the blood pressure remained within normal limits. The clinical complaints of headache, paroxysmal palpitations and night sweats disappeared postoperatively. Although not measured before surgery, the plasma-free metanephrines were negative several days after the tumourectomy.

## Anatomopathological findings

**Figure 4.**
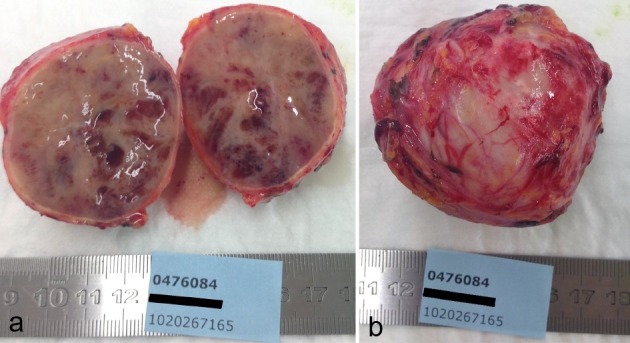
Photographs of the specimen resected at laparotomy shows a firm, spherical mass with a solid surface (a). A midline cross-section (b) shows internal haemorrhagic zones and microcysts. Scale is in centimeters.

**Figure 5.**
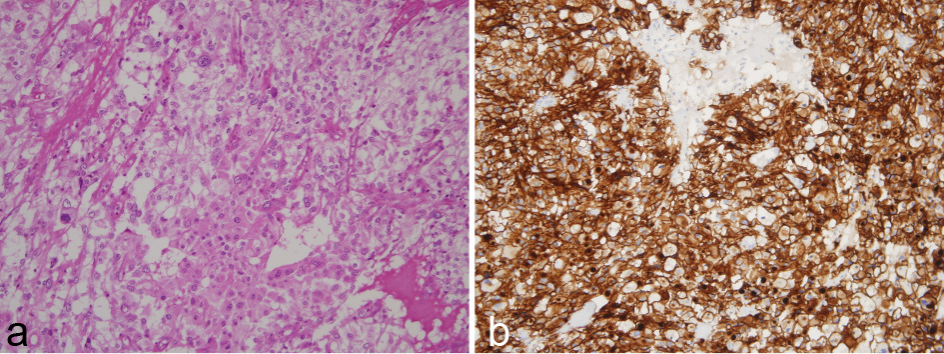
(a) Photomicrograph (×200; haematoxylin and eosin stain) of the mass demonstrates that the tissue has a delicate capillary network and is crossed by numerous vessels. There is more fibrous stroma towards the center. (b) Photomicrograph (×200; CD56 immunostain) showing that the cells are immunoreactive for the neuroendocrine marker CD56, and confirming the diagnosis of a paraganglioma.

Pathology revealed a firm, tan mass, measuring 6 by 6 by 4 cm. Macroscopy showed a solid white surface with haemorrhagic zones and microcysts (Figure 4). The histological structure and immunohistochemical profile matched a paraganglioma, based on the organ of Zuckerkandl (Figure 5).

## Discussion

Paragangliomas are rare neuroendocrine tumours, arising from the paraganglionic system composed of neural crest cells. Tumours that arise from the neuroendocrine cells of the adrenal medulla (80%) are referred to as pheochromocytomas, those that are found in an extra-adrenal location (20%) are called paragangliomas.^1^ Their prevalence has been estimated between 1:6500 and 1:2500.^2 ^

Paragangliomas mainly affect adults in their fourth or fifth decade of life. There is no sex predilection. They may occur isolated or as part of a hereditary syndrome. Up to 30% of patients with a paraganglioma have disease-causing genetic mutations, inherited in an autosomal dominant manner.^3,4^Up to now, 14 different genes have been reported, with different genotype- phenotype presentation and risk of malignancy and recurrence. These familial tumours can also be associated with multiple endocrine neoplasia syndrome and neuroectodermal syndromes; for example., tuberous sclerosis, Type 1 neurofibromatosis and von Hippel-Lindau disease.^5^

The majority of extra-adrenal tumours are seen in specific locations in the neck, chest and abdomen.^5^ A typical location is in the organs of Zuckerkandl, a collection of paraganglia located at the bifurcation of the aorta or at the origin of the inferior mesenteric artery. Forty percent produce high levels of catecholamines, which results in symptoms such as headache, palpitations, excessive sweating and hypertension.^1^ Approximately 10% of the paraganglioma are clinically silent, presenting as an incidental finding.^5^

When there is clinical suspicion for a catecholamine-secreting tumour by physical examination and clinical history, gathering biochemical evidence is recommended. Plasma free metanephrines or 24 hr urinary fractionated metanephrines and catecholamines are measured for the evaluation of catecholamine hypersecretion.

Either CT or MRI is recommended for initial tumour localization. Contrast CT is the primary imaging modality, given its excellent spatial resolution and good sensitivity (88–100%), but as with MRI, it lacks specificity (70–80%).^3 ^ MRI could be a better option in patients with metastatic disease, or when radiation exposure must be limited.

On contrast-enhanced CT scans, a paraganglioma appears as a large well-defined soft-tissue tumour with intense contrast enhancement due to their hypervascular nature. Areas of necrosis may be seen in the tumour, as well as punctate calcifications or focal areas of high attenuation caused by acute haemorrhage. The key to diagnosis is the location of the mass; when a hypervascular mass at the bifurcation of the aorta is found, the possibility of a paraganglioma should always be considered.^5^

On *T*_1_ weighted MR images, these tumours are usually hypointense or isointense compared to liver parenchyma. On spin-echo sequences, signal voids can be seen, resulting in the characteristic “salt and pepper” pattern. On *T*_2 _weighted images, a paraganglioma presents as a markedly hyperintense mass, but the tumour often displays complex and heterogeneous signal intensity due to haemorrhage.^1 ^

Only the presence of distant metastases allows differentiation of malignant from benign paragangliomas on imaging. Patients with large size of tumours, extra-adrenal or recurrent disease have an increased risk for distant metastasis. For detection and follow-up, nuclear medicine is indicated.

Progress in PET imaging allows for reliable early detection of metastatic disease, with a sensitivity of 74–100%.^3,4 ^ Most paragangliomas show uptake of ^18^F-FDG in PET imaging and even benign tumours can be highly ^18^F-FDG-avid.^6^ Aggressiveness and dedifferentiation alone do not explain the high ^18^F-FDG uptake, this in contrast to other neuroendocrine tumours, for example. thyroid cancers and endocrine pancreatic tumours. Specific genetic defects, tumour localization and malignant potential may all play a role in the degree of ^18^F-FDG uptake. However, the exact mechanism for the molecular changes is yet to be determined.

Paragangliomas express somatostatin receptors, enabling imaging with somatostatin receptor PET-tracers such as the ^68^Ga-DOTA-coupled peptides DOTATATE, DOTANOC and DOTATOC. Especially ^68^Ga DOTATATE PET-CT has been shown to have a higher sensitivity and uptake intensity compared to all other imaging modalities, and is a suitable first line investigation when a paraganglioma is suspected.^7^

Microscopically, all paragangliomas have a similar morphological appearance. They are highly vascularised and the cells that produce catecholamines are intimately related to the capillaries. In the presented case, the cells were highly immunoreactive for the neuroendocrine markers CD56, synaptophysis and neuro specific enolase (NSE).

Paragangliomas are more aggressive tumours compared to their adrenal counterparts, with 22–50% versus 2–10% developing metastases.^1^ In up to 10% of patients, metastases are already present at the time of diagnosis.^4 ^ Dissemination occurs via both the lymphatic and haematogenous routes, with the most common sites of metastasis being the regional lymph nodes, bone, liver and lung.^8 ^ The overall 5-year survival rate is 35–60% when metastases are present, with limited treatment options.^4^

Treatment involves surgical excision to reduce the symptoms of excess catecholamine. The decision for laparoscopic or open resection depends on the tumour size and location, as well as on the surgeon’s preference and expertise. Before surgery, patients with a hormonally functional tumour should undergo preoperative blockade to prevent perioperative cardiovascular complications, with α-adrenergic receptor blockers as the first choice.^3^ This emphasizes the role of the radiologist in the preoperative diagnosis, in order to notify the treating physician.

Molecular genetic testing should be considered in all patients with a paraganglioma. Based on the results, decisional algorithms are available for imaging follow-up.^3^ Patients require long-term periodic clinical and imaging follow-up, because metastatic disease or recurrence can appear even after decades free of disease.^4 ^

## Learning points

1. The possibility of a paraganglioma should be considered in the presence of a retroperitoneal, enhancing mass at the bifurcation of the aorta and typical clinical symptoms of hypertension, headache and palpitations.
2. The diagnosis is suggested based on a careful history, physical examination, biochemical and imaging studies.
3. The ^68^Ga DOTATATE PET-CT is a newer and more specific imaging modality, suitable for first line investigation when a paraganglioma is suspected.
4. There is an important role for the radiologist in the preoperative diagnosis, in notifying the treating physician to prevent perioperative catecholamine excess and complications.
5. Treatment involves surgical resection and the final diagnosis is made by pathological examination of the resection specimen.
6. Genetic counselling is not only important to predict the malignancy and recurrence risk, but also for personalized, genotype-based management and follow-up.

## Consent

Written informed consent for the case to be published (including images, case history and data) was obtained from the patient(s) for publication of this case report, including accompanying images.
